# The complete mitochondrial genome of the pallid seahorse *Solegnathus hardwickii* (Actinopterygii; Syngnathiformes; Syngnathidae) obtained using next-generation sequencing

**DOI:** 10.1080/23802359.2018.1502639

**Published:** 2018-08-23

**Authors:** Alireza Asem, Yun-Sheng Xu, Pei-Zheng Wang, Weidong Li

**Affiliations:** a College of Life Sciences and Ecology, Hainan Tropical Ocean University, Sanya, People’s Republic of China;; b Hainan Engineering Research Center of Seafood, Hainan Tropical Ocean University, Sanya, People’s Republic of China

**Keywords:** Mitogenome, pallid seahorse, *Solegnathus hardwickii*, next generation sequencing

## Abstract

The complete mitochondrial genome of *Solegnathus hardwickii* was determined to be 16,519 bp long circular molecule with a typical gene arrangement of vertebrate mitochondrial. The complete mitochondrial genomes were obtained by conventional and long PCR. Tree constructed using maximum likelihood based on protein-coding genes and ribosomal RNAs showed close relationship of *S. hardwickii* with *Hippocampus* spp.

Pallid seahorse *Solegnathus hardwickii* (Gray 1830) is a species of Syngnathidae family that distributed in habitats with hard substrates of Japan, Australia (eastern and northwestern) and South China Sea (Pollom 2017). The species of *Solegnathus* are highly worthwhile in the Asian medicine especially in Chinese traditional medicine (Courtney et al. 2007). Despite their valuable economic importance, there is a lack of information on the biology and ecology of *Solegnathus* species (Courtney et al. 2007). Here, we reported the complete mitochondrial genome of *S. hardwickii* (GenBank accession no. MH539788) to compare its phylogenetic platform with other members of Syngnathidae.

An adult of *S. hardwickii* was collected from South China Sea (Hainan province, China; 19° 02’ N 110° 44’ E) and stored in Hainan Tropical Ocean University Museum of Zoology (NO.0001-Sh). The genomic DNA was extracted from dorsal-lateral muscles (30 mg) using Rapid Animal Genomic DNA Isolation Kit (Sangon Biotech Co., Ltd., Shanghai, CN; NO. B518221). A genomic library was established followed by next-generation sequencing. Quality check for sequencing data was done by FastQC (Andrews 2010) and the fragments sequences were assembled and mapped using SPAdes version 3.9.0 (Petersburg, Russia) (Bankevich et al. 2012).

The complete sequence of *S. hardwickii* was 16,519 bp in size with a base composition of 29.75% A, 15.16% G, 26.73% T, and 28.36% C, including 13 protein coding genes (PCGs), 2 ribosomal RNAs (srRNA and lrRNA), 22 transfer RNAs (tRNAs), and and a control region (CR) of 903 bp. The genes of the *S. hardwickii* mitogenome are in the same order and orientation as in *Doryichthys boaja* mitogenome (Asem et al. 2018).

There was a strong A + T bias (56.47%). The longest gap and overlapping were determined between *tRNA-Asn*/*tRNA-Cys* (38 bp) and *ATP8*/*ATP6* (10 bp), respectively. All PCGs began with common Met start codon, while *COX1* was encoded with Val. Stop codons included TAA (*ND1*, *ND2*, *COX1*, *ATP8*, *ATP6*, *ND4L* and *ND5*), TAG (*ND6*) and incomplete codon T (*COX2*, *COX3, ND3*, *ND4* and *Cytb*). The *12S ribosomal RNA* and *16S ribosomal RNA* were encoded from 71 to 1010 (940 bp) and 1084–2751 (1668 bp), respectively, with 16S having a rather higher A + T content (56.71 vs. 53.83%). These were located between the *tRNA-Phe* and *tRNA-Leu*, and were separated by the *tRNA-Val*. Seven tRNAs (*tRNA-Gln*, *tRNA-Ala*, *tRNA-Asn*, *tRNA-Cys*, *tRNA-Tyr*, *tRNA-Ser*, and *tRNA-Glu*) and just *ND6* protein-coding gene were encoded on the light strand and others were encoded on the heavy strand.

The phylogenetic relationship of *S. hardwickii* among Syngnathidae family was determined from a concatenated dataset including the 2 rRNAs and 13 PCGs using the software MEGA 7.0.26 version (Pennsylvania, USA). (Kumar et al. 2016) with 1000 bootstrap replicates and GTR model (Figure 1). According to the result of phylogenetic tree, *S. hardwickii* was placed as a clade sister to *Hippocampus* spp. It needs to sequence the mitogenomes of other members of Syngnathidae to determine the phylogenetic status among them.

**Figure 1.**
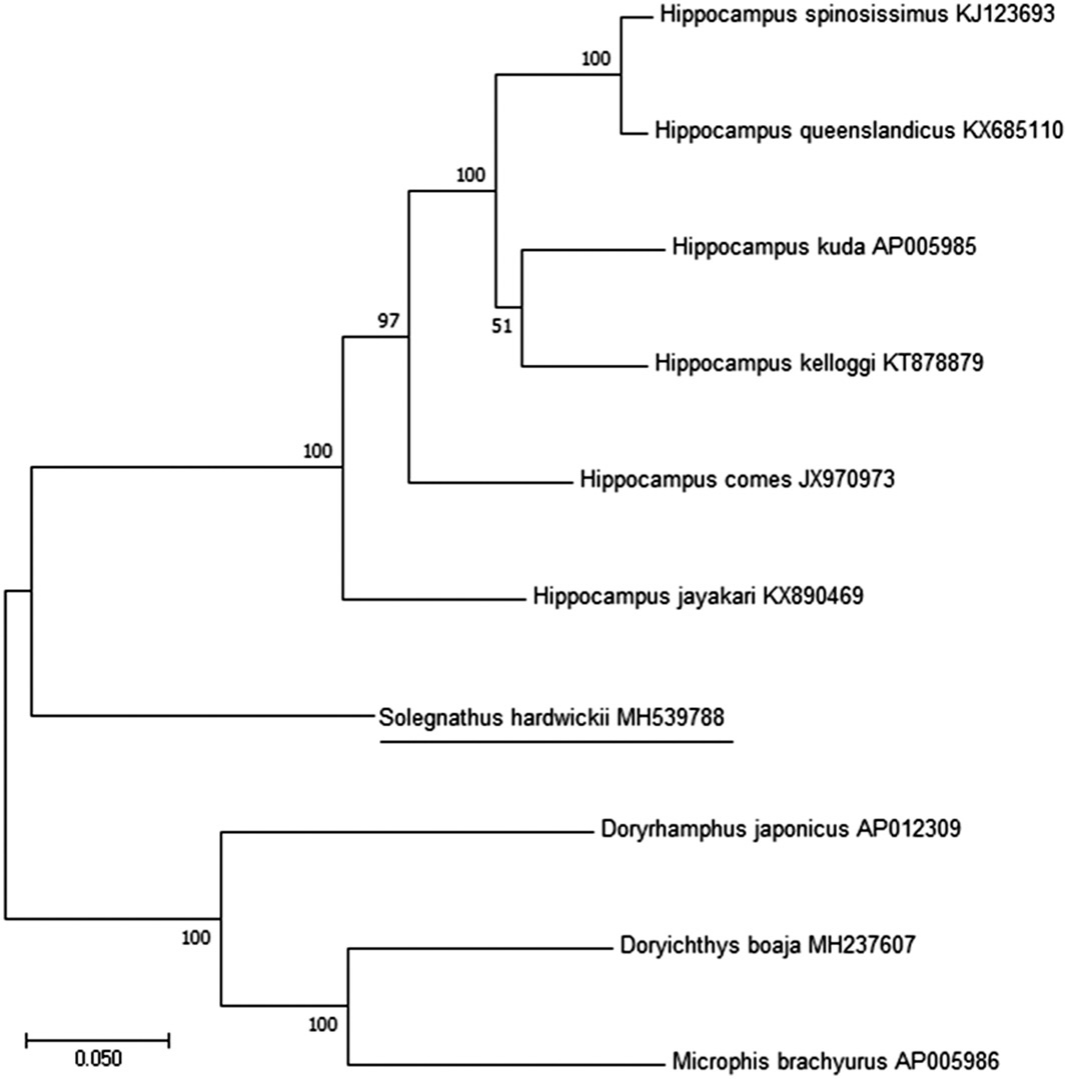
Phylogenetic tree showing the relationship among *S. hardwickii* and nine other species of Syngnathidae based on maximum-likelihood (ML) approach. Numbers behind each node denote the bootstrap support values. The GenBank accession numbers are indicated on the right side of species names.
